# Community-based surveillance of unaccompanied and separated children in drought-affected northern Ethiopia

**DOI:** 10.1186/s12914-019-0203-9

**Published:** 2019-06-10

**Authors:** Matthew MacFarlane, Beth L. Rubenstein, Terry Saw, Daniel Mekonnen, Craig Spencer, Lindsay Stark

**Affiliations:** 1 Department of Population and Family Health, Mailman School of Public Health, Columbia University, 60 Haven Ave, Level B2, New York, NY 10026 USA; 2 Department of Epidemiology, Mailman School of Public Health, Columbia University, 60 Haven Ave, Level B2, New York, NY 10026 USA; 3 Migration Management Unit, The UN Migration Agency (IOM), Special Liaison Office, Addis Ababa, Ethiopia; 4 Brown School at Washington University in St. Louis, One Brookings Drive, St. Louis, MO 63130 USA

**Keywords:** Surveillance, Unaccompanied children, Separated children, Ethiopia, Child protection

## Abstract

**Background:**

Children separated from their caregivers in humanitarian emergencies are vulnerable to multiple risks. However, no field-tested methods exist to capture ongoing changes in the frequency and nature of separation in these contexts over the course of a protracted crisis.

**Methods:**

Recognizing this gap, a mobile phone-based surveillance system was established in a drought-affected district in northern Ethiopia to assess the feasibility of using community focal points to monitor cases of unaccompanied and separated children. A total of 29 focal points were recruited through village elections from 10 villages in the district. Feasibility was assessed directly by measuring the number and quality of messages sent by the focal points each week. The team also evaluated the implementation process and any challenges that arose through observations and key informant interviews with focal points at the conclusion of the project measuring frequency of employing various information gathering techniques, challenges faced, and perceptions of community expectations. Likert scales were used to measure overall satisfaction with the experience of being a focal point, self-assessed difficulty of being a focal point, perceived likelihood of cases captured, and motivation.

**Results:**

Over a six-month period, the focal points reported 48 cases of separation. The majority of separated children (64.6%) were 10 years of age or older. Work was a major driver of separation, especially for boys. Age, sex, role in community, and density of community had no statistically significant impact on focal point performance in terms of frequency, accuracy, or consistency of messages. The focal points themselves reported high levels of motivation, but suggested several areas for improvement in the surveillance system.

**Conclusions:**

Without the surveillance system, most of these children would have otherwise been unrecognized. From a technical standpoint the system was successful and resilient in the face of unexpected external challenges. However, focal point participation and accuracy was variable over time and across groups and diminished towards the later months of the study, suggesting that the community-based approach may require additional supports to ensure that the surveillance system is able to accurately capture trends over time.

**Electronic supplementary material:**

The online version of this article (10.1186/s12914-019-0203-9) contains supplementary material, which is available to authorized users.

## Background

Family separation is one of the greatest protection risks faced by children in humanitarian emergencies. Children can become separated from their parents or customary caregivers due to a multitude of emergency-related circumstances, including caregiver death, migration, poverty and food insecurity [1, 2]. In some cases, separated children are cared for by relatives or other adults. In other situations, separated children do not have any adult caregivers – this latter subgroup of separated children are referred to as “unaccompanied.” In comparison with children who are not separated, unaccompanied and separated children (UASC) experience adverse physical and psychological outcomes, including increased food insecurity, violence, exploitation, and stress [3–5].

In an effort to mitigate these adverse outcomes, there is widespread consensus within the humanitarian field that family tracing and reunification of unaccompanied and separated children should be among the first activities that practitioners implement after an emergency. Standards and best practices to guide these critical activities are well-established [6, 7]. Yet, despite decades of applied programmatic experience, several gaps in understanding unaccompanied and separated children remain, especially regarding information about the frequency and characteristics of separation in a given emergency setting.

Recognizing these gaps, the Child Protection Working Group introduced the Child Protection Rapid Assessment (CPRA) toolkit in 2012 as a way to generate local data about child protection concerns in emergencies, primarily through qualitative interviews with key informants [6, 8]. The toolkit is intended for use six to eight weeks after the onset of an emergency and includes questions about unaccompanied and separated children. In addition, a population-based survey about separation was also recently piloted in the Democratic Republic of the Congo. The survey approach is intended to complement the CPRA by quantitatively estimating the prevalence of child separation following a specific emergency event [9]. However, both the CPRA and the population-based survey tool remain limited by the fact that they only capture information at a single point in time.

The lack of data on trends in the frequency and characteristics of unaccompanied and separated children over time creates significant challenges for all aspects of response within protracted and/or rapidly changing emergency settings. Without a dynamic understanding of the movement of separated children, implementing organizations are unable to target and adapt their programs effectively for maximum impact. An interagency advisory panel therefore came together in 2014 to design a method for measuring separation trends that could be implemented in protracted emergency settings and yield information for both humanitarian responders and advocates.

A community-based surveillance system was proposed as a cost-effective approach to capitalize on local knowledge of children’s movement over a sustained period of time. Community-based surveillance relies on a network of trained community members who relay specific observations about their surroundings to a central body on a regular basis. The method has been used to gather information about topics ranging from infectious diseases to maternal health and nutrition in hard-to-reach places [10–14]. In addition, borrowing from the success of community-based surveillance systems in other resource-poor settings, it was proposed that data be collected through mobile phones [15, 16]. Compared with paper-based reporting, phone-based reporting increases the speed and efficiency of community-based surveillance systems [17].

The first pilot of a community-based surveillance system to measure unaccompanied and separated children was carried out in the conflict-affected region of North Kivu in the Democratic Republic of the Congo (DRC) over an 11-week period in 2014 [18]. Focal point performance in the DRC was variable and questions remained about the ability of the system to operate in diverse contexts for extended timeframes. In 2016, Northern Ethiopia experienced a significant drought and was selected for the second pilot in order to further explore these outstanding issues against the backdrop of a different emergency setting. In describing the findings from the second pilot of the community-based surveillance system, the manuscript aims to (1) assess the overall feasibility of implementing such a system in different contexts, and (2) make recommendations on considerations for similar approaches in the future.

## Methods

### Overview and definitions

A community-based surveillance system was established in the Tigray region of northern Ethiopia in June 2016 with the primary goal of measuring trends in the frequency and characteristics of unaccompanied and separated children over time. A secondary goal of the system was to generate learning to refine the data collection methods and tools in order to conduct similar research in other settings. The surveillance system was developed by methodologists from Columbia University in partnership with Save the Children and implemented with the International Organization for Migration (IOM), as well as in close collaboration with the Government of Ethiopia and the national Child Protection Sub-Cluster.

Unaccompanied and separated children were defined following the UN Convention on the Rights of the Child [19]. Separated children were defined as children who have been separated from both parents, or from their previous legal or customary primary caregiver, but not necessarily from other relatives. Therefore, separated children may include children under the care of other adult family members. Unaccompanied children were defined as children who have been separated from both parents and other relatives and are not being cared for by any adult who, by law or custom, is responsible for doing so.

### Ethics approval and consent to participate

This study was covered under Columbia University Medical Center’s IRB reference AAAQ8815, as well as ethical approval from Save the Children’s Ethical Review Committee, the Ethiopian Ministry of Women’s and Children’s Affairs and an ethics review committee of local experts from outside the government structure. Consent was obtained at three levels: first, in written or oral form from community liaisons, depending on their preference; second, orally from community leaders in a private meeting; and third, communal consent to participate was obtained orally from community members at each site during public meetings. As the project focused on liaisons sharing publicly available information about unaccompanied or separated children and strictly forbid direct interaction with children, the researchers and ethical reviewers agreed that a formal assent or consent process with the children or their parents/guardians would potentially increase risk to those individuals and thus was not included in the procedures. Any information communicated was without identifying information and came from community knowledge that the liaisons would be privy to rather than direct interviews with children or guardians.

The authors acknowledge the financial support of the United States Agency for International Development’s Office of Foreign Disaster Assistance (AID-OFDA-G-15-00176). Statements made in this paper are the views of the authors alone, and do not constitute the policy of the above listed funding bodies.

### Study context

In early 2016, the Ethiopian government and international aid organizations began calling for support from the global community for an impending famine caused by significant droughts throughout the northern part of the country. Large-scale child separation, along with malnutrition and other protection challenges, was predicted to be a consequence of the famine as caregivers became unable to adequately provide support to their children [20, 21].

After extensive consultation with the Ethiopian Ministry of Women’s and Children’s Affairs and other partners, the project team decided to implement the surveillance system in the Adwa district. Adwa is located adjacent to the communities most severely affected by the famine and therefore significant movement of children in this district was expected. Within Adwa, five *kebeles* (administrative units in Ethiopia, usually encompassing approximately 2000 households) were selected for data collection based on estimated scale of separation, accessibility and cell phone network reliability. The research team and the Project Coordinator consulted with the chairperson for each of the selected *kebeles* to seek their consent to proceed with the pilot. All chairpersons agreed to support the study. Subsequently, the project team conducted meetings in ten villages across these *kebeles* to seek general communal feedback and consent to participate in the study. All ten villages agreed to participate.

### Focal point selection process

Following communal consent, each village held an election to choose three focal points per village. The elections were guided by the following criteria: two focal points were elected from the existing village leadership structures and one focal point was elected from outside this structure; at least one focal point in each village had to be a female.

### Reporting protocol

After the focal points were elected, they met with the research team to identify and agree on a clearly defined area of 100 to 200 households within which they would be responsible for reporting cases of separation. In some villages, this reporting area covered a small, relatively densely populated geographic area, while in other villages, it encompassed large fields and farms. By including both types of areas, the project was better able to represent the diversity of conditions in the region. All three focal points in a given village were responsible for reporting on the same pre-defined area of 100–200 houses. Focal points were asked not to work together or share information with one another.

When a focal point learned of a case of separation in his or her reporting area, s/he was asked to send a text message containing a numeric code to the Project Coordinator’s central phone (See Additional file 1 ‘Community Liaison Codebook’). Whenever possible, this six-component code provided all of the following information about each child: 1) age (exact or approximate); 2) sex; 3) whether the child arrived in the community, departed from the community, or was remaining in the same community; 4) whether the child was separated or unaccompanied; 5) reason(s) for the separation; and 6) current caregiver(s). Focal points were required to submit a distinct string of code for every child, even if multiple children were separated by the same incident or were residing together.

To monitor engagement, if focal points did not encounter any new case of separation in the past week they were expected to send a message with the code “0000” to the Project Coordinator on Sundays. If focal points noticed a situation requiring urgent action or had a problem and needed to talk to the Project Coordinator, they were instructed to text “9999” at any time to receive a return phone call as soon as possible. Services for urgent actions and referrals were provided in partnership with existing local response structures, particularly the Ministry of Women and Children’s Affairs.

### Data retrieval and verification

**Fig. 1:**
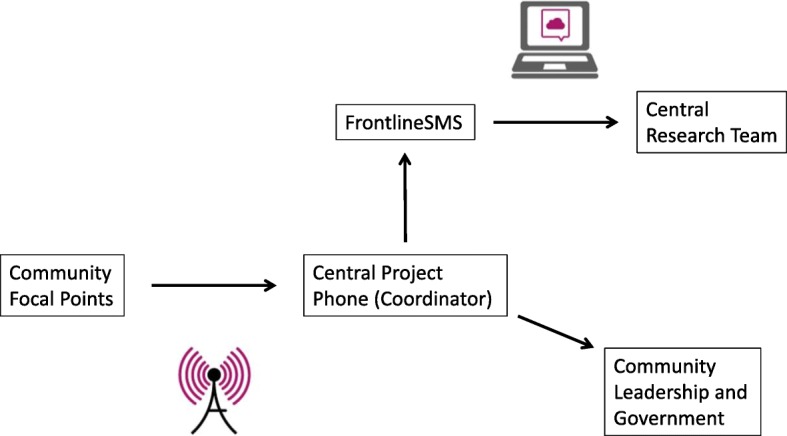
Diagram of the reporting system

All text messages were sent to a central smartphone connected to FrontlineSMS, a free, open-source software that enables automatic transmission of coded text messages to a special web-based inbox. This set-up allowed project and research staff to remotely retrieve and monitor reports from villages (see Fig. 1).

A dedicated IOM Project Coordinator served as the main field agent and point of contact for the focal points. Every time a case report was received via the FrontlineSMS system, the Coordinator called the focal point who had sent the report. On this call, the Project Coordinator verified the reported information (e.g., it was a new case of separation, it occurred within the focal point’s defined surveillance zone, the codes were entered correctly). If any inconsistencies were identified, errors were logged and focal points sent corrected reports. The Project Coordinator also asked the focal point to provide the initials of the separated child in order to cross-check with reports from other focal points to identify duplicate reports about the same child.

### Training

The 30 focal points were invited to a three-day training workshop led by the research team and the Project Coordinator. Of the 30 focal points elected, 29 participated in the full training; one dropped out of the project. The workshop covered several topics, including technical training on the use of the phone, reporting procedures, and how to identify and file urgent action situations. Each topic included multiple practice scenarios and role-plays for both groups and individuals. Participants also engaged in discussions about different ways to identify cases of separation (e.g., active visits to neighbors’ households, compared with passive learning at weekly community gatherings), but focal points were not required to adhere to any specific case-finding method. At the end of the workshop, focal points were given a laminated sheet with the numeric reporting codes, a detailed manual with procedures and scenarios, and a letter to signify their role in the study. All documents were translated into the local language, Tigrinya.

### Supervision visits and final evaluation

For the first two months following the training, the Project Coordinator visited each focal point on a bi-weekly basis to answer questions, confirm cases, and fix any mechanical issues with the phones. After the first two months, the visits were reduced to once per month. The Coordinator was also responsible for replenishing any phone credit used by focal points to send text messages, as well as charging costs for the phones in villages without regular electricity. Focal points received no other remuneration, and were considered volunteers.

At the close of the project, the Project Coordinator and the research team returned to each village to conduct individual in-depth interviews with the focal points and learn about their experiences to provide further information on the feasibility and acceptability of the surveillance approach. Twenty-one focal points participated in these interviews undertaken by a member of the Columbia University research team and a local interpreter (See Additional file 2 ‘Key Informant Interview Guide’). Eight focal points who had originally participated in the program did not participate in the interviews. Of these, four had dropped out at some point during the study after moving out of the original community for work or family reasons. Two others were unavailable at the time of the interviews due to work and childbirth, respectively, while the final two could not be reached during the time allotted for the interviews. All villages had at least one focal point interviewed.

The interview tool was developed based on the final evaluation instrument from the first pilot in DRC. Questions measured frequency of employing various information gathering techniques, types of challenges faced, and perceptions of community expectations. Likert scales were used to measure overall satisfaction with the experience of being a focal point, self-assessed difficulty of being a focal point, perceived likelihood of cases captured, and motivation. A total of 24 open and closed-ended questions were created to explore the experience of focal points, including suggestions on how well the program worked and recommendations for future projects. The interview was administered verbally by a Columbia University researcher with the help of a trained interpreter. Each interview lasted approximately two hours, and was conducted in either the focal point’s homes or in a quiet space away from the community. Notes were taken during the interviews and recorded digitally. Translations that were captured in the audio recordings were reviewed and verified by an independent fluent Tigrinya speaker who was external to the study. This check confirmed the accuracy of the translations, which were used to fill out the field researcher’s notes.

### Analysis

Descriptive analyses were performed on a month-by-month basis for total cases of separation from July through December, as well as stratified analyses by children’s sex, age, caregiver status, reason for separation and whether the child was departing from the community, arriving into the community or moving within the community.

To assess system feasibility, three measures were calculated: frequency, accuracy, and consistency. First, frequency of engagement was assessed for any message sent, both correct and incorrect, across the entire 26-week period. Focal points who sent messages in at least 75% of weeks were considered to have “good” reporting frequency, and focal points who sent messages in at least 90% of weeks were considered to have “high” reporting frequency. Accuracy, operationalized as the percentage of case reports that were correctly formatted, was also analyzed to assess the level of understanding of the reporting structure among focal points. Finally, reporting consistency was measured based on the extent to which the focal point sent correctly coded messages of any kind each week (correct messages were defined as a case report, an urgent action request, or a no-case/0000 report). These benchmarks were then used to assess the relative risk of consistent reporting according to various focal point characteristics (specifically sex, age, pre-existing leadership role, and community layout). The performance of different groups of focal points was compared using Chi Square tests at a significance level of *p* < 0.05.

Focal point experiences were assessed based on the in-depth interviews. Data from open-ended questions were coded manually using printed copies of the transcripts and margin notes. A codebook was created using a deductive approach. A data display was constructed to aid in the development of themes, and was based on a simple partially-ordered model using the frequencies from the closed-ended questions to guide the structure. Using the data display, key themes were identified.

## Results

### Child separation

**Fig. 2:**
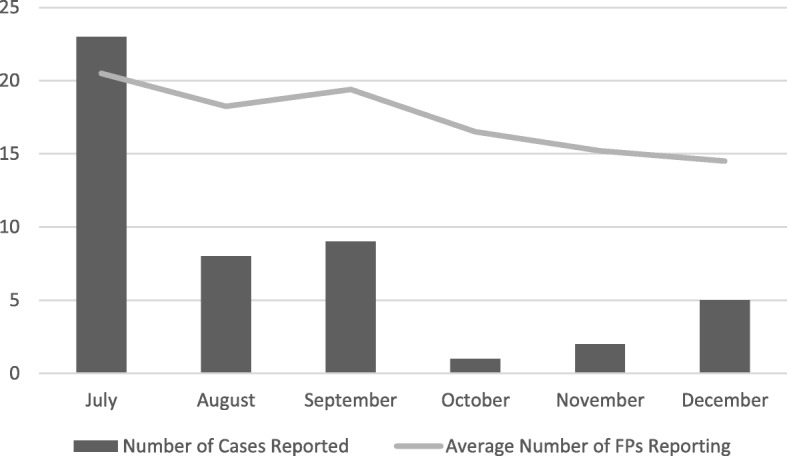
Cases and Focal Points Reporting by Month

Over the course of the six-month study period, 48 individual cases of unaccompanied and separated children were reported and verified (see Table 1). Of these 48 cases, 43.8% (*n* = 21) were children who had arrived to the participating communities, 18.8% (*n* = 9) were children who had become separated within these communities, and 37.5% (*n* = 18) were children who had departed from these communities. Nearly 40% (*n* = 19) of all separated children were reported to be unaccompanied. Distribution of reporting varied across months, ranging from one case in October to 23 cases in July. As illustrated in Fig. 2, most cases of separation occurred in July and August. There was also a wide range in number of cases of separation per village, with one village reporting no cases throughout the study period and another village reporting more than ten cases. Only two duplicate cases were reported over the course of the study.

**Table 1:** Characteristics of UASC by month

	Month							
	JUL	AUG	SEP	OCT	NOV	DEC	Total	%
Sex								
Male	15	7	4	1	1	4	32	66.7
Female	8	1	5	0	1	1	16	33.3
Direction								
Arrived	6	6	4	0	2	3	21	43.8
Departed	11	1	5	1	0	0	18	37.5
Same community	6	1	0	0	0	2	9	18.8
Age								
0–4 years	2	1	1	0	0	1	5	10.4
5–9 years	1	2	2	0	0	2	7	14.6
10–14 years	9	1	1	1	2	2	16	33.3
15–17 years	7	4	4	0	0	0	15	31.3
Don’t know	4	0	1	0	0	0	5	10.4
Status								
Separated	12	5	5	0	2	5	29	60.4
Unaccompanied	11	3	4	1	0	0	19	39.6
Current Caregiver								
Uncle/aunt/grandparent	5	3	2	0	0	5	15	31.3
Sibling	4	0	0	0	0	0	4	8.3
Other family	0	1	2	0	0	0	3	6.3
Non-family adult	0	1	1	0	1	0	3	6.3
Other children	7	1	4	1	0	0	13	27.1
Alone	3	1	0	0	1	0	5	10.4
Don’t know	4	1	0	0	0	0	5	10.4
Reason								
Death of Parents	10	3	2	0	2	0	17	35.4
Work	8	4	4	1	0	0	17	35.4
Guardian Disappearance	0	1	0	0	0	5	6	12.5
Lack of Food	4	0	1	0	0	0	5	10.4
School	0	0	1	0	0	0	1	2.1
Ran away	0	0	1	0	0	0	1	2.1
Other	1	0	0	0	0	0	1	2.1

With regards to the characteristics of the children, there were significantly more cases of separated boys, compared with girls (66.7% vs. 33.3%, p-value = 0.02). This difference was driven in part by a surge of eight adolescent boys who left their communities during the month of July to look for work, more than half of the male separation in that month. The majority of separated children (64.6%) were 10 years of age or older. Among the separated children younger than ten years of age, 11 out of 12 children were newly arrived in their community or became separated within participating communities. The most common reasons for separation were work (35.4%), death of parents (35.4%), guardian disappearance (12.5%) and lack of food (10.4%). Work was a more common cause of separation in boys, compared with girls (RR = 2.33, *p*-value = 0.088) and nearly all the children separated for work reasons were over 10 years of age (most were older adolescents).

Out of the 29 separated children who were reportedly living with an adult caregiver, 60.0% were living with a grandparent, aunt, or uncle (*n* = 25) and 16.0% were living with an adult sibling (*n* = 4). Among the 19 unaccompanied children, 72.2% were living with other children (*n* = 13) and the remaining 27.8% were living alone (*n* = 5). Unaccompaniment was disproportionately concentrated in children ten years of age or older, compared with younger children (RR = 5.42, *p* = 0.023).

### Focal point performance

Due in part to short-term travel and other personal reasons for non-participation, the average number of focal points reporting during a given week was 17 out of 29 (see Fig. 2). Twenty-seven focal points remained resident in their communities for the duration of the study, while two left for a major town for work in late July and September, respectively.

#### Frequency

Although more than half of the 29 focal points had “good” frequency of sending a correct message in at least 75% of the weeks over the six-month study period, only 21% met the “high” level of frequency, sending a message 90% of the weeks or more. The probability of reporting at the “high” level was not significantly different when comparing age groups, sex, or the population density of the area. Focal points elected from the general community performed better than those selected from the existing leadership structure at a level approaching, but not reaching, statistical significance (RR = 5.67, *p*-value = 0.063).

#### Accuracy

Among the 17 focal points who sent at least one case report of a child, the majority (53%) sent correctly formatted reports 100% of the time. Only 3 focal points submitted case reports with less than 50% accuracy. While male focal points demonstrated a higher rate of sending correct reports, there was no statistically significant difference between sexes. The same was true when comparing age, community density, and role in the community.

#### Consistency

Finally, to evaluate overall performance, frequency and accuracy were combined to assess consistency of reporting both regularly and correctly. Seven out of 29 focal points (24%) sent a correct message in at least three quarters of the study’s 26 weeks. Among these, there was no statistically significant difference across focal points when comparing their role in the community, age, or community density. While female focal points had a better level of performance in this metric (42% of women reaching the threshold compared to just 12% of men) the difference approached but did not reach the required level of statistical significance (RR = 3.54, *p*-value = 0.064). Interestingly, over the course of the study, only two duplicate cases were reported by multiple focal points reporting on the same area and population.

### Technical feasibility

There were several technological challenges over the surveillance period which the research team was able to address. These challenges included several broken mobile phones that needed to be replaced, as well as the FrontlineSMS server being unexpectedly blocked throughout Ethiopia in October. The latter challenge was addressed through manual compilation by the Project Coordinator of the reports from focal points, a possibility that had been planned for from the outset and required no changes on the part of the focal points themselves. Although all communities had strong cellular network service at the beginning of study, two villages lost their network tower after three months. As a result, focal points in these villages walked several kilometers outside of the community to send a message or receive calls. While the surveillance system was able to remain operational in face of these challenges, it is possible that some aspects of the quality and reliability of the data collected for assessing the system may have been affected.

### Focal point’s experience

At the completion of the study, the average overall satisfaction rating from the 21 focal points who participated in exit interviews was 8.43 on a scale where 1 indicated “very bad” and 10 indicated “very good.”, with scores ranging from 5 to 10. Respondents highlighted learning new skills, acquiring new knowledge, and having a sense of contributing to their community as reasons for their positive experience. Focal points also enjoyed that their responsibilities did not interfere significantly with other daily activities, such as travel and work. They were told that as volunteers they were not expected to stop their normal activities. One focal point explained why this integrated approach was positive:

“Because it doesn’t affect my personal business…in order to go smoothly, I made a good arrangement of my personal business and this CFP [community focal point role], because of this I felt motivated” – Respondent 6

Negative experiences were mainly tied to practical issues, particularly around network reliability.

“I don’t know, it’s the telecommunications company’s issue. There is some problem with the network, we should go to other places in order to find a signal” – Respondent 21

No focal points reported any adverse reactions or safety concerns based on their participation in the project.

### Focal point motivation

Motivation for participating in the study was derived from the feeling of contributing to the community and helping children in need. As one focal point summarized:

“In order to make those separated children, in order to keep their safety, there might be an exploitation in this community if someone is unaccompanied. He might be faced some exploitation…So after a while, if you pass this to the government, or to other respective authorities, they might take some actions on those separated, on those unaccompanied children, and this keeps my motivation” – Respondent 2

Those who rated their overall experience more negatively pointed to the fact that they were unable to find any cases or did not see any external follow-up for cases that they did find. One focal point explained:

“This should be action-oriented. We need to see action on this. Not just theoretically, but we need action for those cases. I picked medium one (rating) because at first I was very devoted to work as a focal. But after I see some problems from this project [referring to not getting information on what happened to the cases], I felt careless at that time, and from then onwards, my experience start to deteriorate, and that make my experience medium” – Respondent 15

When asked about how motivation could be better maintained or improved in the future, several focal points mentioned that they would like to be compensated financially, either directly or indirectly through allowances from attending further trainings.

“We focal points, we need some help [from the project]. As we have been working for free, or voluntarily from (inaudible), if they make some arrangements about salary, we will be motivated” – Respondent 5

### Community expectations

The focal points interviewed also expressed strong notions that, at least initially, their communities expected some form of assistance in relation to the project.

“Yes, [the community] has expectations. The community they asked me if there is anything this report brings. The asked me every day. They have an expectation to bring assistance to those cases reported” – Respondent 11

Some community members also resented the benefits that they perceived were accruing to the focal points, but sensitization about the voluntary nature of the focal point role helped assuage some of these concerns:

“When we were coming from the first focal point’s house to this house, the people that were gathering around there, they were having a gossip that I would get some benefit, and partially the community members didn’t understand my role. I wore this [new clothing] last week and after they see this new stuff, they gossip that ferengi [foreigner] has bought him these stuff, so after they see you, they say that ferengi has bought him these stuff” – Respondent 15

“At first [the community] had some misunderstanding because they expected some assistance, but later after we gave them some awareness regarding [our role], they understand that we are serving as a volunteer to report those cases” – Respondent 1

## Discussion

The experience of this community-based surveillance pilot in Ethiopia generated important learning about how to establish an effective and sustainable surveillance system in a resource-limited setting. The system was resilient from a technical standpoint, able to respond to unexpected and expected logistical challenges primarily due to the resourcefulness of focal points and the project’s built-in plans for such issues. The biggest challenge to feasibility of such a system was the sustained involvement and motivation of the community focal points. In Ethiopia as well as the DRC, the most significant predictor of consistent focal point reporting was whether or not a focal point was elected from outside the existing leadership structure. These data strongly suggest that future surveillance systems should encourage participation from community members who do not already hold local leadership positions, and who may have more motivation and sustained interest for the work. Female focal points in Ethiopia also performed better than males, but this finding was not as significant as the contrast between existing leaders and general members of the community. Furthermore, the system appears to function equally well regardless of the area’s population density.

The specific outcomes on child separation from this pilot have important implications for local programming and policy. First, despite widespread concern about high levels of separations in Ethiopia due to drought and famine, during the study period, lack of food was cited as the primary reason for separation in only five cases. In contrast, work was the primary reason for separation in 17 cases. Second, the experience of separation in Ethiopia appears to be highly gendered, with clear intersections between gender and work. Overall, there were significantly more separated boys than girls. Boys were also more likely than girls to be unaccompanied and also more likely to be separated in order to find work. Moreover, older adolescent boys made up the largest group of children during the peak of separations in July. Alternative, community-based income-generating options or the provision of support services in industrial and agricultural hubs may benefit adolescent boys likely to migrate for seasonal work in Tigray region and similar settings.

The findings also have broader methodological implications for community-based measurement of separated and unaccompanied children. Most focal points in Ethiopia had a difficult time categorizing children living with extended family members as “separated.” This tendency for focal points to underreport separated (accompanied) children likely explains the unusually high proportion of unaccompanied children in the sample (40%). Research in Rwanda found that community members often define separation differently than the way it is operationalized by international actors [22]. It is critical to keep these differences in mind when interpreting the results of community-based surveillance systems. Whenever possible, international actors should make efforts to adapt their definitions to reflect local customs and norms.

### Limitations

The most serious limitation of the community-based surveillance system was related to focal point motivation. Surveillance systems operate under the assumption that focal points’ level of participation is consistent for the duration of data collection, but in both Ethiopia and the DRC, focal points’ involvement waned over time. During their exit interviews, focal points in Ethiopia indicated that their motivation would be improved if they could link identified cases of separation to better response services. There are certainly legitimate ethical reasons to provide strong referral mechanisms for cases. Still, even when these mechanisms were provided in DRC, focal point participation also could not be sustained at consistent levels for the duration of the pilot [18]. Anecdotal reports in Ethiopia suggested that the demands of the harvest season were a particularly burdensome interference for many focal points.

Additionally, the extent to which the surveillance system completely captured all cases of separation in the designated monitoring areas is questionable. Despite the fact that there were three focal points responsible for observing the same geographic areas and the focal points were requested to work independently, only two duplicate cases were reported over the entire six-month pilot. This low number of duplicates likely implies that the reported cases are not exhaustive. However, it is still possible for a surveillance system to accurately monitor trends in the frequency and characteristics of separations over time without being comprehensive if focal points’ level of participation remains consistent. Therefore, efforts to improve surveillance system quality should prioritize incentives that encourage consistent participation and potentially consider a smaller target area for each focal point to oversee.

The study had several limitations, both in assessing the feasibility of the surveillance system and the measurement of UASC. First, the small sample size of only 29 focal points meant that comparisons across groups suffered from limited power, particularly in comparing the 17 focal points who ever sent a case report. From a technical standpoint, the blockage of FrontlineSMS in Ethiopia midway through the project meant that all reports needed to be manually entered, which may have resulted in missed or incorrectly recorded entries which could have affected focal point assessment. While the in-depth interview process was valuable for gathering the experience and viewpoints of the focal points, there was a missed opportunity to interview all focal points and other stakeholders, particularly the Project Coordinator; future assessments of similar systems might usefully include this. Finally, unequal network and device challenges across villages and individuals could have affected individual focal points differently, altering their capacity to send messages and their motivation and thus affecting their performance and possibly comparisons across groups.

## Conclusion

There is increasing interest among practitioners to have simple, low-cost methods to better identify separated children in emergencies. The findings described here from a six-month pilot in Ethiopia indicate that community-based surveillance using mobile phones did successfully identify cases of unaccompanied and separated children who may otherwise go unrecognized. Despite some technical challenges, it was possible to implement the system in a resource-limited setting. However, the data should not be interpreted as demonstrative of trends in the frequency and characteristics of unaccompanied and separated children over time. Focal point participation was variable and diminished towards the later months. Future iterations of community-based surveillance systems to monitor separated children must address this key limitation. Electing focal points from outside the existing leadership structure or choosing female focal points are two strategies that show the potential to help with this issue. It is also worth exploring alternative incentives such as increased remuneration. Without improvements to the consistency of focal point participation, community-based surveillance should be considered a case-finding mechanism, rather than a tool to monitor trends.

## Additional files

Additional file 1: Community Liaison Codebook. a. Tool for community focal points to summarize publicly known information about a specific unaccompanied or separated child in text message. (PDF 351 kb)

Additional file 2: Key Informant Interview Guide. a. Tool for interviewer to gather comments and feedback from community focal points at the end of the study period. (PDF 168 kb)

## Data Availability

Due to ethical considerations, the dataset used for this manuscript cannot be shared with readers. The study location is clearly identified within the manuscript and it is likely that someone familiar with the local context may link the data to specific villages and focal points, even without traditional identifying information. In conjunction with the highly sensitive nature of identifying child separation within a humanitarian setting, data-sharing may raise serious privacy and protection concerns. In addition, during the informed consent process, the subjects did not authorize disclosure of the study data. Therefore, any change to the data sharing policies at this time is both unethical and unauthorized. The data also cannot be released without prior IRB approval.

## Abbreviations

CFP: Community focal point
CPRA: Child Protection Rapid Assessment
DRC: Democratic Republic of the Congo
IOM: International Organization for Migration
UASC: Unaccompanied and separated children
