# Supplementary material for: Community-based surveillance of unaccompanied and separated children in drought-affected northern Ethiopia
Source: BMC Int Health Hum Rights. 2019 Jun 10;19:19. doi: 10.1186/s12914-019-0203-9 (PMC6558760; doi:10.1186/s12914-019-0203-9)
Supplement: Supplementary file 1 — Community Liaison Codebook. a. Tool for community focal points to summarize publicly known information about a specific unaccompanied or separated child in text message. (PDF 351 kb) [file 12914_2019_203_MOESM1_ESM.pdf]

**Step 1**		**.**	**Step 2**		**.**	**Step 3**		**.**	**Step 4**		**.**	**Step 5**		**.**	**Step 6**		**.**
Age	*Code*		*Sex*	*Code*		*Separated or Unaccompanied?*	*Code*		*Departed or Arriving Child?*	*Code*		*Reason for Separation?*	*Code*		*He/she lives with whom?*	*Code*	
Provide Age	00-17	.	Female	20	.	Separated	30	.	Disappeared/departed from community	40	.	Water shortage	50	.	Parents	70	.
0-4	94	.	Male	21	.	Unaccompanied	31	.	New arrival in community	41	.	Death of parents/primary caregivers	51	.	Uncle/aunt/grandparent s	71	.
5-9	95	.	Don't Know	28	.	Don't Know	38	.	Neither departure nor arrival (in same community)	42	.	Work	52	.	Brothers/sisters > 17 years	72	.
10-14	96	.							Don't Know	48	.	School	53	.	Other family >17 years	73	.
15-17	97	.	**If no cases occurred during the week. send a message with 0000 indicating you have nothing to report**<br><br>**If you need someone to call you. send a message with 9999**									Lack of food	54	.	Non-family other adults	74	.
Don't Know	98	.										Ran away	55	.	With other children	75	.
• **You must send at least one text message per week by Sunday of each week to 0935802756**<br>• **For each child, provide a code for all six steps. even if you do not know the answer for each step**<br>• **Each step must be separated by a period**<br>• **If there are multiple responses within the same step, you may write up to 2 responses separated by a comma. Example: 05. 20. 30.42.50,51.60**												Loss of shelter/property	56	.	Spouse	76	
												Marriage	57	.	Institution/group home	77	.
												Lost during movement	58	.	Alone	78	.
												Guardian disappearance	59	.	Other	79	.
												Loss of livestock	60	.	Don't know	88	
												Trafficking/kidnapping	61	.			
												Other	62	.			
												Don't Know	68	.			
