# Supplementary material for: Community-based surveillance of unaccompanied and separated children in drought-affected northern Ethiopia
Source: BMC Int Health Hum Rights. 2019 Jun 10;19:19. doi: 10.1186/s12914-019-0203-9 (PMC6558760; doi:10.1186/s12914-019-0203-9)
Supplement: Supplementary file 2 — Key Informant Interview Guide. a. Tool for interviewer to gather comments and feedback from community focal points at the end of the study period. (PDF 168 kb) [file 12914_2019_203_MOESM2_ESM.pdf]

Date: __ __ / __ __ / __ __ __ __

Official Title: ___________________________ Administrative Level: ________________________

Kebele: _____________________________ Site: _____________________________

1. How did you hear about this pilot project?
a. I was told about this pilot by the MSiE team (i.e. Columbia University, Save the Children, IOM).
b. From Federal Government Administration.
c. From the Zonal office.
d. From the Woreda Office.
e. From Kebele Chairperson/Manager.
f. From Community Focal Points.
g. Other(s) (please specify): __________________________________

2. What did you understand about the purpose of the pilot project?

____

____

3. Based on your own experience and what you previously knew about separated and unaccompanied children, do you think this is an issue of concern? Please explain your reason(s).

____

____

4. Has your opinion changed now that the project is over?

Yes ☐ No ☐

IF YES, GO TO 4A. IF NO, SKIP TO Q5.

4a. Please explain why you changed your opinion:

____

____

5. In your opinion, do you think it is useful to measure separation of children? Please explain the reason(s) for your answer.

____

____

6. Based on your previous experience, what do you think are the top three reasons for separation of children?

____

____

6a. Now that the pilot program has ended, based on what you learned during the course of the pilot, have your opinions of the top three reasons for separation changed? Please describe how:

____

____

7. On a scale from 1 to 10 where 1 is not likely and 10 is very likely, how likely do you think it is that Community Focal Points learned about all of the new cases of unaccompanied and separated children in their village / children who left their village?

0	1	2	3	4	5	6	7	8	9	10
Not Likely					Maybe Likely					Very Likely

7a. Please describe why you chose the answer above:

____

____

8. On a scale of 1 to 10, where 1 is Very Bad, and 10 is Very Good; overall, what was your perception of the Measuring Separation in Emergency Situations project?

0	1	2	3	4	5	6	7	8	9	10
Very Bad					Neither Good or Bad					Very Good

8a. Could you please provide the reason(s) for your answer above?

____

____

9. What could be done to improve a monitoring program like this in the future?

____

____

10. Do you think a monitoring program like this would be useful for your organization/government to adopt in the future?

Yes ☐ No ☐

10a. Please describe the reason for your answer below:

____

____

11. What was your experience working with the MSiE team throughout the duration of the project? Please elaborate:

____

____

12. Were there any concerns that arose with regards to the project or with some of the cases that were identified from the project?
If yes, please describe:

____

____

13. Do you have any other questions or comments?

____

____

Additional Questions for Kebele Chair persons and/or Kebele Managers.

1. How much time did you spend in your role to support the MSiE project?

__ __ hours per week

IF ‘0’ HOURS IS MENTIONED, SKIP TO Q2.

2. Could you please briefly describe your role in supporting the MSiE project?

____

____

3. Did you work with the Community Focal Points in the geographic area you oversee to report cases?

Yes ☐ No ☐

If yes, please explain how:

____

____

4. Did any of the Community Focal Points approach you for any reason relating to the project?

Yes ☐ No ☐

IF YES, GO TO 4A. IF NO, INTERVIEW ENDS HERE.

4a. Why did Community Focal Points approach you? Did they…

i.	… need technical assistance with using the cellphones,	Yes	No	DK
ii.	… need clarification on how to report the cases,	Yes	No	DK
iii.	… need to report an urgent action case,	Yes	No	DK
iv.	… just wanted to talk to you casually,	Yes	No	DK
v.	… want to find out about cases from you, or	Yes	No	DK
vi.	… have any other reason?	Yes	No	DK

If yes, please state what other reason(s): ____

____

5. Based on your experience during the past 6 months, how many children do you think were separated or unaccompanied (either arrived, departed, or no movement) in the geographic area of the pilot program?

__ __ in total.

5a. Out of that number, how many do you think on average were separated or unaccompanied in a week/month (depending on the number)?

__ __ per week / month.

6. Do you have records of the children that are separated and unaccompanied?

Yes ☐ No ☐

7. Which month(s) do you think had the most number of separated and unaccompanied children? Could you please explain the reason(s) for your answer?

____

____

8. Do you think this happened because of the on-going drought, or is this an annual occurrence? Please provide an explanation for your answer.

____

____
